# Multinational Tagging Efforts Illustrate Regional Scale of Distribution and Threats for East Pacific Green Turtles (*Chelonia mydas agassizii*)

**DOI:** 10.1371/journal.pone.0116225

**Published:** 2015-02-03

**Authors:** Catherine E. Hart, Gabriela S. Blanco, Michael S. Coyne, Carlos Delgado-Trejo, Brendan J. Godley, T. Todd Jones, Antonio Resendiz, Jeffrey A. Seminoff, Matthew J. Witt, Wallace J. Nichols

**Affiliations:** 1 Centre for Ecology and Conservation, University of Exeter, Cornwall Campus, Penryn, Cornwall, United Kingdom; 2 Biology Department, Drexel University, Philadelphia, Pennsylvania, United States of America; 3 SEATURTLE.org, Durham, North Carolina, United States of America; 4 Instituto de Investigaciones sobre los Recursos Naturales, Universidad Michoacána de San Nicolas de Hidalgo, Morelia, Michoacán, Mexico; 5 NOAA Fisheries, Pacific Islands Fisheries Science Center, Honolulu, Hawaii, United States of America; 6 Instituto Nacional de Ecología, Dirección General de Vida Silvestre, Secretaria de Medio Ambiente y Recursos Naturales, Ensenada, Baja California, Mexico; 7 NOAA—National Marine Fisheries Service, Southwest Fisheries Science Center, La Jolla Shores Dr., La Jolla, California, United States of America; 8 California Academy of Sciences, Golden Gate Park, San Francisco, California, United States of America; Monash University, AUSTRALIA

## Abstract

To further describe movement patterns and distribution of East Pacific green turtles (*Chelonia mydas agassizii*) and to determine threat levels for this species within the Eastern Pacific. In order to do this we combined published data from existing flipper tagging and early satellite tracking studies with data from an additional 12 satellite tracked green turtles (1996-2006). Three of these were tracked from their foraging grounds in the Gulf of California along the east coast of the Baja California peninsula to their breeding grounds in Michoacán (1337-2928 km). In addition, three post-nesting females were satellite tracked from Colola beach, Michoacán to their foraging grounds in southern Mexico and Central America (941.3-3020 km). A further six turtles were tracked in the Gulf of California within their foraging grounds giving insights into the scale of ranging behaviour. Turtles undertaking long-distance migrations showed a tendency to follow the coastline. Turtles tracked within foraging grounds showed that foraging individuals typically ranged up to 691.6 km (maximum) from release site location. Additionally, we carried out threat analysis (using the cumulative global human impact in the Eastern Pacific) clustering pre-existing satellite tracking studies from Galapagos, Costa Rica, and data obtained from this study; this indicated that turtles foraging and nesting in Central American waters are subject to the highest anthropogenic impact. Considering that turtles from all three rookeries were found to migrate towards Central America, it is highly important to implement conservation plans in Central American coastal areas to ensure the survival of the remaining green turtles in the Eastern Pacific. Finally, by combining satellite tracking data from this and previous studies, and data of tag returns we created the best available distributional patterns for this particular sea turtle species, which emphasized that conservation measures in key areas may have positive consequences on a regional scale.

## Introduction

Effective management of large marine vertebrate species is complicated as they often undertake migrations passing through waters of multiple states with varying legislation, social and religious customs [1–3] or through international waters where management of fisheries is ineffective due to logistical complexity [4]. It is evident that anthropogenic impacts remain the major threat to marine vertebrates. Fishery activities are the main cause of marine vertebrates decline [5], be it by intentional hunting [4,6] or incidental capture by artisanal and industrial fisheries [5–9]. Currently, five of the seven sea turtle species are considered endangered or critically endangered [10]. This status is the result of human impacts such as incidental capture by fisheries [11], poaching of eggs and adults [12,13], habitat loss, and pollution [14,15]. Moreover, climate change has recently been identified as a possible threat for sea turtles [16,17]. Considering that this group of marine vertebrates spend the majority of their life at sea, understanding their migratory strategies and habitat use is therefore necessary to implement effective management strategies [18,19].

In recent years satellite tracking has been extensively used to study the movements of sea turtles [20], this has allowed scientists to progress from descriptive movements of turtle migration to interdisciplinary approaches [21]. Through this research, insights have been gained into the spatial ecology of sea turtles, including habitat use in foraging grounds [22,23], locating migration corridors [18,24], identifying strong site fidelity displayed by some species across multiple years [25,26] and their ecological importance within threatened ecosystems [27,28]. These findings demonstrate the need for international cooperation in sea turtle conservation [29]. Telemetry studies have also highlighted likely interactions between sea turtles and fisheries [30–32] and have helped to characterise spatial efficacy of marine protected areas (MPAs) [21,33].

The East Pacific green turtle (*Chelonia mydas agassizii*), also known as the black turtle, is a distinct group with different characteristics from green turtles elsewhere [34]. They inhabit tropical and sub-tropical waters within the eastern Pacific [35] spanning from southern California, USA [36,37] to Chile [38–42] and the Galapagos Islands [38]. Major rookeries in Mexico are found in Michoacán [43–46] and Islas Revillagigedo Archipelago [39,47,48]. In Michoacán, 2362 females are estimated to nest annually at Colola (18° 18’ N; 103° 25’W) and Maruata (18° 16’ N; 103° 20’ W) beaches [46]. Although intensive monitoring has not been carried out on the Islas Revillagigedo, Holroyd & Trefry [48] recorded 500 nests on Isla Clarion (18° 26’N; 114° 52’W) during a two-week period. Two other major rookeries have been described in the eastern Pacific; the Galapagos Islands [38,49,50] where a total of 2756 females were tagged on 4 nesting beaches during 2002 [49], and the Pacific coast of Costa Rica [51], at Cabuyal (10° 40’N; 85° 39’W) [52] and Isla San José (10°51′N; 85°54’W) beaches [53] and Nombre de Jesús beach (10° 23’ N; 85° 50’W) where approximately 15 turtles nest per night during peak season [54].

Flipper tag returns [35,38,45,55], and genetic studies [56] have offered preliminary insights on distribution patterns of east Pacific green turtles (hereafter referred to as green turtles). Tag return and genetic studies offer the valuable contribution of distribution and point to point information but do not explain the migratory paths followed by individual turtles. In recent decades use of satellite telemetry data to describe migratory paths and behaviour at sea during the post-nesting period has increased greatly. Findings suggest that some turtle populations migrate vast distances as part of their life histories [20,50,57]. However due to the sustained and combined threat faced from artisanal [58] and industrialised fisheries throughout the eastern Pacific where lack of regulation and management is widespread [59], more knowledge is needed on the movements between breeding and foraging areas in order to highlight possible migratory corridors used by turtles. Resources for ocean conservation in this vast region are generally lacking or limited making obtaining detailed information about the distribution of endangered species critical to directing available funds and efforts as they become available. Here, we intended to analyse migration patterns and movements within and between foraging grounds of East Pacific green turtles and nesting beaches in southern Mexico. Additionally, we used existing data from other rookeries along the eastern Pacific to contextualise distributional patterns of turtles during migration and on both foraging and breeding grounds relative to the threats faced due to human activities. In particular we, (1) assess movements of turtles from the Michoacán rookery using satellite telemetry, (2) describe patterns of movement on the northern foraging grounds of Baja California, (3) analyse movements at a wider spatial scale by integrating our findings with previous flipper tagging data and information from other satellite tracking studies.

## Materials and Methods

Research was authorized by the *Secretaría de Medio Ambiente*, *Recursos Naturales y Pesca* (permit numbers 150496–213–03, 280597–213–03, 190698–213–03, 280499–213–03) and the *Secretaría de Medio Ambiente y Recursos Naturales* (permit number SGPA/DGVS/002). All animal handling was in full compliance with IACUC protocol at the University of Florida.

We deployed satellite transmitters (see below) on 12 green turtles (9 female; 3 male) between 1996 and 2007 (see Table 1 for details). It is important to mention that two of the turtles had spent time in captivity at *Centro Regional de Investigación Pesqueras-Estación de Conservación e Investigación de Tortugas Marinas* (CRIP-ECITM) Baja California (in January 1997 and November 1998). Nine of the 12 turtles tracked were captured on the foraging grounds off the coast of Baja California and an additional three turtles were tracked from the Michoacán nesting rookery. From those 12 tracked turtles, three post-nesting adult females were tagged at Playa Colola in the state of Michoacán during the months of February and March of 2001. An additional seven adult-sized turtles (3 male and 4 female) were tracked on their foraging grounds at Bahia de los Angeles (n = 4), Bahia de Loreto (n = 2), and near Isla San Jose (n = 1), and the remaining two turtles were tagged and released from captivity (Table 1).

**Table 1:** Summary data of morphometrics and tracking details of 12 East Pacific green turtles.

Turtle	SCL^1^ (cm)	Sex^2^	Period in Captivity	^3^Release location	Released	Mean speed (km h ^-1^)	Locations <-200m (%)	Distance travelled (km)	Final location	Duration (d)
A	75.6	F	10.5 y	BLA	25-Jan-97	1.27	71	2928	Nesting	108
B	89.9	F	<1d	Loreto	28-Aug-97	1.40	57	1337	Nesting	57
C	74.3	F	2.3 y	BLA	23-Nov-98	1.47	70	1932	Nesting	57
D	78	F	<1d	Colola	02-Mar-01	1.08	85	941.3	Foraging	48
E	-	F	<1d	Colola	14-Feb-01	0.83	64	3020	Foraging	196
F	66.6	F	<1d	Colola	27-Feb-01	0.94	49	2927	Foraging	153
G	89.2	M	<1d	BLA	23-Jul-97	0.54	57	13.7	Foraging	21
H	91.8	M	<1d	BLA	21-Jul-04	0.62	-	691.6	Foraging	
I	72.5	M	<1d	Near Isla San Jose	03-May-07	1.28	-	206.0	Foraging	53
J	88.7	F	<1d	BLA	04-Aug-97	0.04	100	17.4	Foraging	9
K	77.3	F	<1d	BLAP	04-Aug-97	1.09	67	329.7	Foraging	23
L	80	F	<1d	Loreto	11-Aug-97	0.08	100	51.1	Foraging	15

^1^SCL: Straight carapace length.

^2^F: Female; M: Male.

^3^BLA: Bahía de los Angeles; BLAP: Bahía de los Angeles Park.

### Capture and Tagging Procedure

Turtles in foraging areas were captured using entanglement nets. Nets were 100 m long by 8 m high with a stretched mesh size of 50 cm and set to capture turtles at foraging locations known to have been historically productive areas for turtle fisheries. Nets were monitored every 2 hours to ensure the safety of captured turtles [60]. For all turtles, satellite transmitters deployed were Telonics ST-6 Platform Transponder Terminals (PTTs) (Mesa, Arizona, USA, n = 7) that weighed 500 g in air; Wildlife Computers SDR-SSC3 PTT that weighed 750 g (n = 3), one Wildlife Computers Spot-5 with one C-cell that weighed 350 g; (Seattle, Washington, USA), and one Kiwisat with 2 D cells weighing 825 g (Havelock, New Zealand). Satellite transmitters were attached to the second vertebral scute of the turtle’s carapace following the methodology described by Balazs et al. [61] with modifications as described by Nichols [60]. Silicone Elastomer was used to attach the transmitter to the carapace, and marine epoxy (Marine-Tex; Montgomeryville, PA, USA) was used to create a faring as described by Watson & Granger [62], to improve hydrodynamics and to reduce the impact of tags on the turtles [63] caused by drag [64].

For the purposes of this study, locations (latitude and longitude) were the only information utilized from the satellite transmitters.

### Movement Analysis

Argos location data were filtered using Satellite Tracking and Analysis Tools STAT [65]. STAT provided data on seafloor depth at each location received from the PTTs. We plotted turtle movements using ArcView 9.2 GIS software (Environmental Systems Research Institute, Inc). To avoid excluding useful data, positions from six valid location classes (3, 2, 1, 0, A, B) were used. The data were then filtered to remove biologically unrealistic results; speeds greater than 5 km h ^-1^, and changes in swimming direction of an angle of <25° [66]. Two pseudo reference points were inserted into the migration routes of Turtles B and C (see Table 1) so that the track followed the coast, rather than cross land, in the vicinity of the Bahia de Banderas. To classify post-nesting movements of adult females, we used a 4 point classification framework described by Godley et al [20]. Type A1—oceanic and/or coastal movements to neritic foraging grounds: where migration routes are generally direct, but may include coastal sections that increase migratory distance; Type A2—coastal shuttling between summer foraging and wintering sites: where turtles move towards areas of warmer water temperatures during the winter season; Type A3—local residence: where turtles remain in the vicinities of the nesting areas migrating very short distances; or Type B—Pelagic living: Turtles spend most of their time in pelagic waters with occasional use of neritic environments [20].

Additionally, satellite tracking methods were supplemented with data on sea turtle distribution from flipper tags obtained from a review of existing literature (detailed in S1 Table). These data were used to obtain a more complete scenario of the movements and a possible overlap in the distribution of individuals from different breeding sites in the eastern Pacific, in order to contextualise the East Pacific green turtle meta-population.

### Threat analysis

Data of Halpern et al. [67] detailing the cumulative global human impact (fisheries, pollution, invasive species, climate change, ocean acidification, nutrient input, human population pressure and commercial activities (shipping)) on marine ecosystems were used to give insights into the relative threat faced by East Pacific green turtles during their migrations. To do this, tracks were interpolated to 1 km distances between locations. Each resulting shapefile was then used to sample the value of the underlying Halpern et al. [67] data using the extension Hawth’s Tools for ArcGIS. For each location in the Halpern et al. [67] shapefile the spatial coincident values were taken for comparison with the overall neritic threat level, which was calculated by sampling the area of ocean up to 200 km from the Mexican Pacific coastline. The threat faced by turtles while on their foraging and nesting grounds was also calculated from the Halpern et al. [67] raster. Each start or end point of a turtle’s track was classified as either foraging or nesting depending on the area in which it was located. To compare threats faced by Michoacán turtles with those of the Galapagos and Pacific Costa Rican populations, the end points of were taken from Seminoff et al. [50] study of the Galapagos population and Blanco et al. [57] study of the Pacific Costa Rican population. An area, described by a radius of 20 km was then sampled around each point from the values on the Halpern et al. [67] raster using Hawth’s Tools Zonal Statistics (++). A median threat index was then calculated. To access threat level at each of the four major green turtle rookeries (Michoacán, Galapagos, Pacific Coast of Costa Rica and Islas Revillagigedo) a central location from each rookery was taken and the threat was calculated using the above methodology except that the area sampled was increased to 100 km radius.

## Results

### Adult female migration

**Fig 1:**
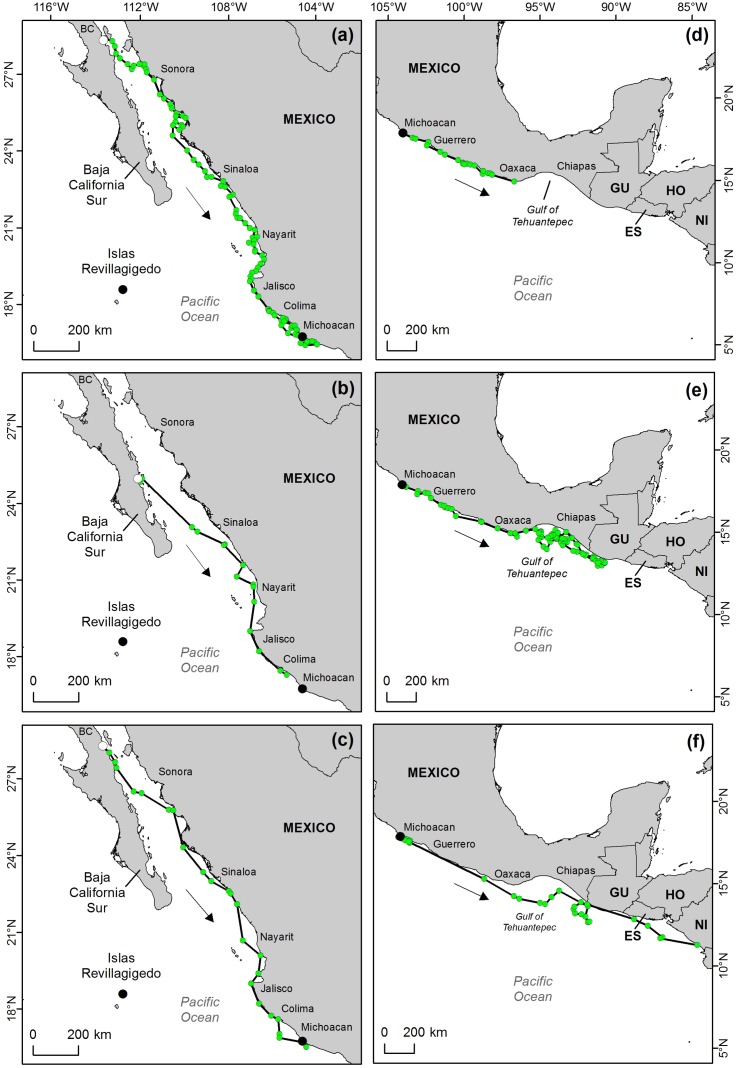
Migratory routes taken by female East Pacific green turtles. (a, b, and c) migrating from their foraging grounds in Baja California (BC) and Baja California Sur to their breeding grounds in Michoacán. Turtle A and B were tracked during 1997 and turtle C during 1998–1999. White circles indicate the start point of tracks (d, e, and f). Post-nesting migration from Michoacán during 2001 of three turtles (turtles D, E and F respectively) to the southern foraging grounds in Central America.

Adult females A, B and C (see Table 1) were tracked from their foraging grounds on their southward migrations towards the Michoacán breeding grounds for 108, 57, and 57 days respectively (Fig. 1 a, b, c). All turtles performed Type A1 migrations, departing from neritic foraging grounds following the coast to reach the nesting rookeries. Turtles A (Fig. 1a) and C (Fig. 1c) departed from the foraging grounds of Bahia de los Angeles, Baja California within 1–4 days after deployment. These two turtles covered a minimum straight-line distance of 2694 km (turtle A) and 1743 km (turtle C) towards the breeding grounds in the state of Michoacán. Transmissions continued to be received from turtle A for 36 days after arrival to the vicinities of the nesting beach of Colola, Michoacán, during which time looping behaviour was displayed, moving up to 29 km offshore. On the contrary, transmissions from turtle C continued to be received for only 6 days after her arrival to waters off the nesting beaches. Turtle B, remained in the foraging grounds (Bahia de Loreto) for approximately 24 days before exiting the bay covering a minimum along-track distance of 1207 km (Fig. 1b). Transmission ceased in the waters of the state of Colima, approximately 97 km north of the nesting beach of Colola in Michoacán. The routes taken by these turtles were reasonably direct. All kept to the coastal shelf except for the necessary crossing of the Gulf of California from the Baja California peninsula to mainland coast of Mexico (See Fig. 1). Mean travel speed of these pre-nesting turtles was 1.4 ± 0.1 km h ^-1^ (range 1.3 to 1.5 km h ^-1^) but when crossing the Gulf of California mean speed increased to 1.7 ± 0.7 km h ^-1^ (range 1.5 to 2.3 km h ^-1^).

Arrows indicate direction of tracks. MX: Mexico; GU: Guatemala; ES: El Salvador; HO: Honduras; NI: Nicaragua

Adult females D, E, and F (see Table 1) were tracked from their nesting beach in Colola, Michoacán southwards to their foraging grounds in Central America (Fig. 1 d, e, f). The three turtles presented “Type A1” migratory patterns: coastal movements to neritic foraging grounds [20], moving along the coast as they travelled south. Mean travel speed of these turtles was 0.95 ± 0.1 km h ^-1^ (range 0.8 to 1.1 km h ^-1^) which was generally slower than individuals moving towards breeding sites. Turtle D started its journey at Colola beach (Fig. 1d) migrating 902 km southward. Transmissions ceased in coastal waters in the vicinity of Oaxaca, Mexico (15°35’ N 96°32’ W), suggesting transmitter failure or turtle capture. Turtle E (Fig. 1e) travelled a minimum straight-line distance of 2915 km following the coastline of the Gulf of Tehuantepec, before heading into deeper waters in front of the Gulf of Tehuantepec where the turtle displayed looping behaviour for the remaining 99 days of transmission (see Fig. 1e). Turtle F (Fig. 1f) travelled 2829 km point of origin. During that journey, the turtle deviated from its coastal movements along the north Oaxaca coast before entering the Gulf of Tehuantepec and then regaining the coast near north Guatemala. Southward movement continued until 153 days after deployment when transmissions ceased in southern Nicaraguan coastal waters.

### Foraging movements

Six turtles tracked from their foraging grounds (G-H, Table 1) remained in the area during the study period. For the 3 males (G, H, I) and 3 females (J, K, L) that remained on the Baja Californian foraging grounds (See Table 1, S1 Fig.) tracking durations were short (25 d ± 15.3 d, range = 9 to 53 d), however data were sufficient to highlight a number of aspects. Although there appeared to be a degree of fidelity to the foraging area, this was not extreme, with animals ranging a maximum of 13.7 to 691.6 km from point of release at mean speeds of 0.60 h ^-1^ (range 0.04 to 1.28 km h ^-1^ see Table 1). All turtles tracked by satellite remained within shallow coastal areas. Briefly, adult male (G) and female (J) remained in Bahia de los Angeles, Baja California (S1A Fig.); whereas, foraging female (L) moved within Bahia de Loreto, Baja California Sur (S1B Fig.). Interestingly, one foraging male (turtle H) moved southwards out of Bahia de los Angeles before returning to its release location displaying looping behavior (S1C Fig.); one adult female (turtle K) moved out of Bahia de los Angeles into the north of the Gulf of California (S1D Fig.); finally, turtle I remained near the foraging areas of Isla San Jose and Isla Partida, Baja California Sur (S1E Fig.).

### Contextualising movements within the meta-population

**Fig 2:**
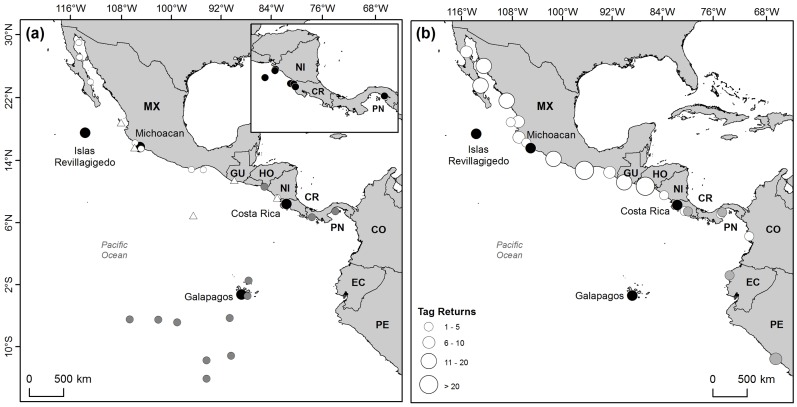
Distribution of adult green turtles in the Eastern Pacific Ocean. Black circles show the locations of nesting sites at the four major East Pacific green turtle rookeries: Michoacán, the Galapagos Islands, Islas Revillagigedo and the north Pacific Coast of Costa Rica. (a) Distribution of ending positions of green turtles satellite tracked from Michoacán nesting grounds: this study (white circles n = 12) and Byles et al. [71] (white triangles, n = 5,). Grey circles represent end points from turtles tracked from the Galapagos Islands (n = 12; Seminoff et al. [50]). Inset map: end positions for turtles tracked from Costa Rica and represented by grey circles (n = 9; Blanco et al. [57]). (b) Number and location of tag returns from turtles flipper tagged in Michoacán = white circles (Alvarado & Figueroa [43]; Marquez & Carrasco [45] Zavala pers comm; Llamas pers comm) and the Galapagos Islands = grey circles (Green [38]). Circles are scaled to the range of tag returns for each region. Fig. 2 (b) does not include the 44 tag returns that were reported within the state of Michoacán. MX: Mexico; GU: Guatemala; HO: Honduras; NI: Nicaragua; CR: Costa Rica; PN: Panama; COL: Columbia; EC: Ecuador; PE: Peru

Satellite tracking studies from Michoacán (present study), Galápagos [50] and Costa Rica [57] showed a wide distribution of East Pacific green turtles through Central American waters (Fig. 2a).

Overall 218 flipper tag recaptures from Mexico and 23 recaptures from Galapagos were obtained from the literature (S1 Table, Fig. 2b). That information suggested that turtles from the Michoacán population are distributed from the Baja California Peninsula (Mexico) to Colombia. Whereas turtles from the Galapagos population migrate to Central and South American coastline, with tag return data being collected from Honduras to Peru [38,43,45,50]. Although, there is insufficient published data on the green turtle population of the Islas Revillagigedo, as a result of flipper tag studies this population is known to be distributed from the Islas Revillagigedo north to San Diego Bay (USA) [39].

The combination of satellite tracking and tag return studies suggested that there is considerable overlap of the Michoacán, Galapagos and Costa Rican populations foraging areas in Central America (Fig. 2 a, b), as tracking studies of the Pacific Coast of Costa Rica green turtle population (Fig. 2a inset), showed that adult females also remain on the Central America foraging grounds after nesting [57].

### Threat assessment

The putative anthropogenic threat faced by East Pacific green turtles was assessed using a dataset of integrated human impact described by Halpern et al. [67] (S2 Fig.). We assessed the threat faced by East Pacific green turtles at each of the four major rookeries (Michoacán, Islas Revillagigedo, Galapagos and Costa Rica). Overall, this analysis suggested that the Costa Rican rookery presented the highest threat level (mean 8.08 ± 3.75) when compared to the nesting grounds in Mexico (Michoacán and Islas Revillagigedo) where the threat level was medium, and with those in Galapagos which presented the lowest threat level (3.5 ± 3.1). Of the two Mexican rookeries, Michoacán presented a slightly higher level of threat (6.3 ± 5.5) than the Islas Revillagigedo (6.0 ± 1.8).

**Fig 3:**
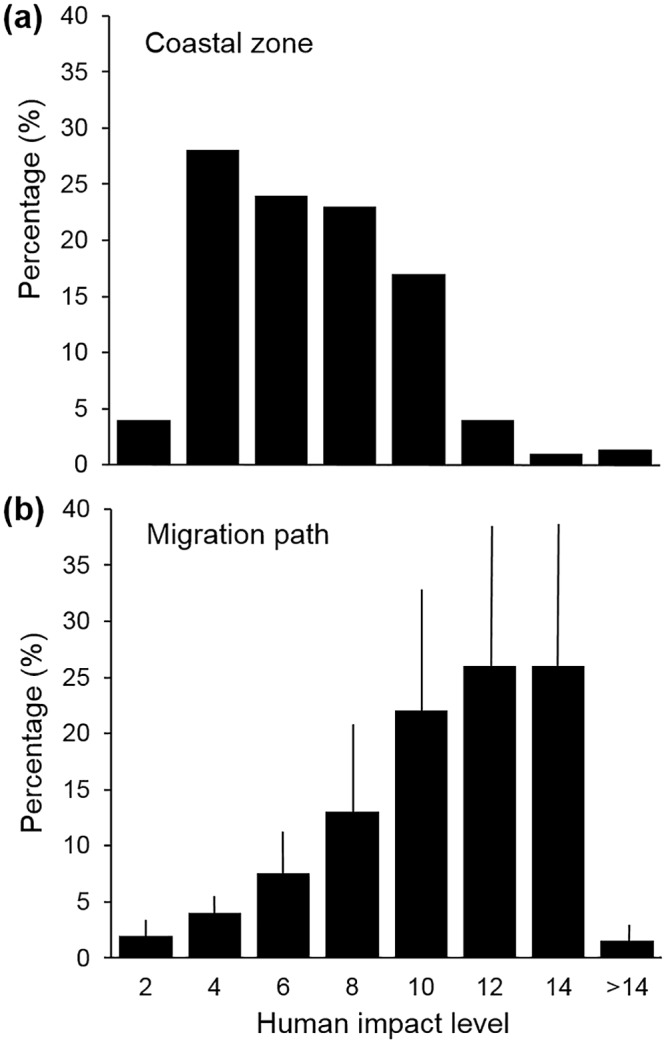
Threat level faced by East Pacific green turtles. (a) Histogram comparing the percentage of each threat level faced in the coastal area to the (b) actual average threat level faced by six migrating East Pacific green turtles (turtles A-F) migrating from Baja Californian foraging grounds to breeding grounds in Michoacán (n = 3) and from Michoacán breeding grounds to Central American foraging grounds (n = 3)

Migrating turtles from this study (Mexican population) were found to spend a greater amount of time in areas of higher impact and therefore may be more threatened than would be expected based on the average threat level within the Mexican Pacific coastal area (200 km from coast) (Fig. 3). When we considered only foraging locations, turtles from the Costa Rican population foraged in areas of highest impact (8.6 ± 2.8, n = 9). The Michoacán foraging turtles were found to forage in areas of higher impact (7.2 ± 1.8, n = 10); than those from the Galapagos population (6.0 ± 3.43, n = 10); however, the difference was not found to be significant (t-test, t13.53 = 0.98, p = > 0.05).

## Discussion

Understanding the complex interactions of animals from different reproductive stocks is key to the implementation of successful regional conservation measures [68]. The present study represents the first attempt to describe the distribution of East Pacific green turtles *(Chelonia mydas agassizii)* throughout their range combining novel and existing data on turtles from three major rookeries (Michoacán, Galapagos and Pacific coast Costa Rica) including composite information collected during multiple years of sea turtle monitoring along the coast of the Eastern Pacific.

### Adult female migration

Turtles from the Michoacán rookery travelled extensive distances (max. 2,694 km) between breeding and foraging grounds. Those tracked on migrations towards nesting beaches maintained a mean travel rate of 0.45 km h ^-1^ which was faster than individuals leaving nesting sites towards foraging grounds. This difference in swimming speed may suggest females forage along their post breeding migration. Swim speeds were similar to rates recorded for other populations within the Eastern Pacific: Galapagos Islands (0.8 km h ^-1^ [50]), Pacific Costa Rica (1.5 km h ^-1^ [57]) and green turtles (*Chelonia mydas*) from other regions: coastal movements in the Mediterranean (1.6 km h ^-1^ [69]) and Brazil (1.0 km h ^-1^ [70]).

Green turtles migrating from northern foraging grounds to Michoacán nesting areas displayed Type A1 coastal migrations and were only observed leaving coastal waters during the necessary crossing of the Gulf of California from the Baja California peninsula to the mainland. The displayed migratory behavior following the coastline was also displayed by post nesting females migrating to southern foraging grounds, although, when two of three individuals migrated through the Gulf of Tehauntepec, they moved into oceanic waters. This coastal migratory behavior is similar to that displayed by some of the post-nesting turtles migrating from Costa Rica [57]. None of the turtles tracked in this study displayed behaviour suggestive of oceanic foraging as seen in most of the Galapagos green turtles [38] and in one Michoacán turtle described by Byles et al. [71]. Despite of the limited sample size, we believe that for adult females nesting on the continental mainland, oceanic foraging may be a minority foraging strategy. Seminoff et al. [50], suggested that oceanic foraging may be more energetically beneficial than neritic foraging for turtles nesting on Galapagos Islands. It might be possible that for turtles nesting on the continental mainland (as the Michoacán rookeries) may be less costly in terms of energy to migrate and forage along the coast. Although telemetry data are lacking at present, it would be interesting to learn and compare if a greater prevalence of offshore foraging is present for green turtles nesting at the Islas Revillagigedo, the other major offshore archipelago in the region.

### Foraging turtles

Despite the relatively short number of days foraging turtles were tracked during this study we were able to observe local movements of six individuals. As previously shown using satellite telemetry [57,72], the distances travelled within foraging areas suggested that green turtles have large home ranges. Adult male turtle H tagged in Bahia de los Angeles travelled 691.6 km displaying a typical looping behaviour suggestive of foraging [27,73]. Although traveling a greater distance than other foraging *Chelonia mydas*, this male returned to the point of release before transmission ceased, which implies that the turtle was not beginning migrational movements. Additionally, that type of movement suggested that this turtle may be resident to Bahia de los Angeles. Turtle K also travelled a considerable distance of 329.7 km moving into the northern Gulf of California. Although turtle K did not display typical looping behaviour we considered movements to be representative of foraging as there are no registered nesting rookeries for green turtles in the northern Gulf of California, and the area is well documented as a foraging area for this species [58,74,75,76]. Large foraging ranges of green turtles may be a result of low food densities within home ranges and their diverse diet having been found to feed on seagrass, algae and invertebrates [74,77] compared with turtles from other *Chelonia mydas* populations [78]. Their diverse diet allows them not to be limited to environments of shallow sea grass beds. Large foraging areas have also been shown for green turtles feeding on algae in Brazil [70] and East Pacific green turtles foraging along the Nicaraguan coast [57].

### Contextualising movements within the meta-population and threat assessment

Green turtles from both the Michoacán (this study) and Galapagos rookeries [50] have been shown to migrate to foraging grounds in Central America, and, thus far, the Costa Rica population has been shown to remain in this region during non-reproductive life phases as well [57]. Supporting these telemetry studies information obtained from tag recaptures (see S1 Table) demonstrated that green turtles tagged in Mexico, were recaptured in Central (Guatemala, El Salvador, Nicaragua, Costa Rica, Panama, and Ecuador) and South American countries (Colombia and Peru), the same occurred with turtles tagged in Galapagos Islands which also were recaptured in Central and South American countries. This may indicate that green turtles from this distinctive group along the Eastern Pacific show high interaction between individuals congregating in the same foraging areas despite originating from distant nesting rookeries. For example, turtles nesting on Mexican beaches are present in the foraging grounds (i.e. Nicaraguan) with individuals from Galapagos and Costa Rican nesting beaches. This is also supported by genetic studies suggesting that the genetic composition of turtles foraging in Gorgona National Park, Colombia comprise haplotypes that have been found in turtles from Mexico and Galapagos [79]. Recently, Dutton et al. [80] indicated that the Costa Rican rookery is genetically closely related to the Galapagos rookery while turtles from Michoacán are differentiated from those two rookeries despite of the mixing in the foraging grounds. Several studies focused their attention on the origins of sea turtle migration [81]. As suggested by Blanco [82], foraging areas of green turtles nesting on Costa Rican beaches are the result of hatchling dispersal with ocean currents. This is supported by Scott et al. [81], where authors indicate that the diverse migration routes of adult sea turtles reflect the variety in neritic and oceanic areas they may have known during their pelagic phase as drifting hatchlings. Therefore, we could suggest that the major foraging areas for this species located along the coast of Central America are a result of hatchlings from Mexico, Galapagos, and Costa Rica being transported by oceanic currents to those areas. Green turtles were found to be at risk from anthropogenic threats throughout their breeding migrations and feeding ranges. Turtles from all three rookeries were found to migrate towards Central America whereas only turtles from the Michoacán rookery were seen to move further north into the Gulf of California. This considerable overlap of foraging areas added to the fact that Costa Rican populations (foraging in Central America) are at higher risk because of human activities (see results), highlights the importance of the Central American coastal areas to the survival of these remaining major East Pacific green turtle populations. Green turtle density in the Central American region is supported by findings of high levels of green turtle bycatch by Costa Rican shrimp trawlers [83,84]. In addition there are several artisanal long line fisheries operating along the coast of Costa Rica. These fisheries are not controlled when fishing outside a protected area; therefore those fishing vessels are not accounted for by any agency (Blanco personal observation). As a consequence, sea turtle bycatch may be much higher than official reports suggest as some artisanal fisheries have been shown to have bycatch levels similar to that of industrial fisheries [85].

Although not demonstrated extensively by our data, green turtle populations may be threatened by fishing activity due to the pelagic foraging habits of some individuals as shown by Seminoff et al. [50] for the Galapagos population and Byles et al. [71] for the Michoacán population. These pelagic habits, though not represented in turtles satellite tracked in this study, are further supported by reports from observers on tuna purse seiners sighting a total of 2736 green turtles between 1993–2001, 54 of which were dead making this species second only to *Lepidochelys olivacea* for observed abundance [40,84,87]. This supports the call for bycatch mitigation to be focussed in the eastern Pacific as an area of high bycatch [59,86].

Our results also highlight the fact that migrating green turtles from Mexican nesting beaches occupy for the most part areas of no specific conservation status (see http://www.conabio.gob.mx/). In 1998 a Mexican government initiative highlighted 70 areas within Mexico’s Exclusive Economic Zone (EEZ) to be known as Marine Priority Areas. This designation was given to highlight areas of high biodiversity, important resources or as a result of a need for research into their biodiversity. (http://www.conabio.gob.mx/). We suggest that by using these Priority Areas as a guide, MPAs could be quickly implemented or seasonal fisheries closures could be applied along the coastline depending on the time of the year when green turtle migrations are taking place. If these Priority Areas were designated MPAs East Pacific green turtles would be fully protected in the Baja Californian foraging areas and in many areas south of the Gulf of California including the Gulf of Techautepec, a possible green turtle foraging ground. However until this happens, the status of these areas as Priority Areas affords no effective protection.

Four of the twelve satellite tracked turtles showed early loss of satellite transmitters. Although we are unable to definitively attribute this to a particular factor, previous studies have suggested reasons for early transmitter loss. Hays et al. [88] identified exhaustion of batteries, salt-water switch failure, antenna breakage, animal mortality and premature detachment of tags as reasons for early loss of transmission. Further Hays et al. [88] attributed loss of transmissions from four olive ridley turtles (*Lepidochelys olivacea*) to their habit of lodging themselves between rocks and corals to rest resulting in damage or loss of the transmitter. This habit of using coral reefs and rocky areas for resting has been documented for green turtles [77,79]. However the main reason for low retention times for satellite telemetry studies on green turtles ([51] Arauz unpublished data, Amorocho unpublished data), is the turtle’s oily carapace and thin keratin layer which may affect tag attachment.

Despite this early transmitter loss we suggest that satellite tracking of foraging green turtles is extremely important in terms of studying the spatial ecology of this species. Other methodologies such as radio telemetry, though highly effective while tracking within bays and lagoons, does not allow tracking of turtle movements with such large home range as the ones described in this study. In addition, the use of flipper tags on sea turtles offers point to point information, but does not describe movements within a foraging area. The use of these techniques may lead to foraging strategies and areas, such as the ones described here, to be overlooked when planning conservation schemes and designing effective MPAs.

We have shown how satellite tags demonstrate a major advantage over flipper tags as they can provide us with information on how individual turtles move between their foraging and nesting grounds allowing us to identify key migratory routes and new foraging areas. We also draw attention to the benefits of satellite tracking of green turtles on their foraging grounds due to the large area of their home ranges. We have also highlighted how traditional flipper tag studies have and continue to contribute to our knowledge of sea turtle movements, can reinforce satellite telemetry and have the added benefit of being economically accessible to many researchers. By combining all data available we can begin to understand the biogeography of widely distributed species.

Additional research and monitoring is necessary to clarify threats faced by green turtles along their migrational routes, such as fisheries interactions and direct illegal harvest. Knowledge of the Michoacán turtles’ movements from the rookeries to foraging areas in Baja California also needs further attention and pelagic movements of this population have yet to be described in detail. Research on the Michoacán green turtle population is not proportional to its regional importance nor to the threat that it continues to face on foraging grounds. We recommend an increase in research into the green turtle on their southern foraging grounds and tracking of turtles leaving Islas Revillagigedo as a complement to existing telemetry studies from the three rookeries presented here. This is the first study to highlight how turtles from three major green turtle rookeries mix on foraging grounds in Central America and we suggest that these Central American foraging grounds should be priority areas for protection and research. This study highlights the importance of expanded conservation efforts in the coastal area of Central America as protection of foraging green turtles at these locations would have positive consequences at a regional scale.

## Supporting Information

S1 Fig Movements of six East Pacific green turtles in the foraging grounds of the Baja Californian peninsula. (a) movements of adult male Turtle G (Solid line) and an adult female (Dashed line) Turtle J in Bahia de los Angeles, Baja California. (b) Adult female Turtle L moving within the Bahia de Loreto, Baja California Sur. (c) Movements of adult male Turtle H moving out of Bahia de los Angeles to the south before returning to the area of its release. (d) Adult female Turtle K moving out of Bahia de los Angeles into the north of the Gulf of California and (e) adult male Turtle I foraging in the area of Isla San Jose and Isla Partida, Baja California Sur.
(TIF)

S2 Fig Map of data from Halpern et al. (2008) detailing the cumulative global human impact on marine ecosystems. Black circles represent major green turtle rookeries in the East Pacific.
Cumulative impacts: fisheries, pollution, invasive species, climate change, ocean acidification, nutrient input, human population pressure and commercial activities (shipping).
(TIF)

S1 Table Summary table of flipper tag returns by region. Flipper tag returns for turtles from the Michoacán, Alvarado & Figueroa [43]; Marquez & Carrasco [45] and Galapagos, Green [38] populations.
(DOC)
