# Supplementary figures and images for: Multinational Tagging Efforts Illustrate Regional Scale of Distribution and Threats for East Pacific Green Turtles (Chelonia mydas agassizii)
Source: PLoS One. 2015 Feb 3;10(2):e0116225. doi: 10.1371/journal.pone.0116225 (PMC4315605; doi:10.1371/journal.pone.0116225)

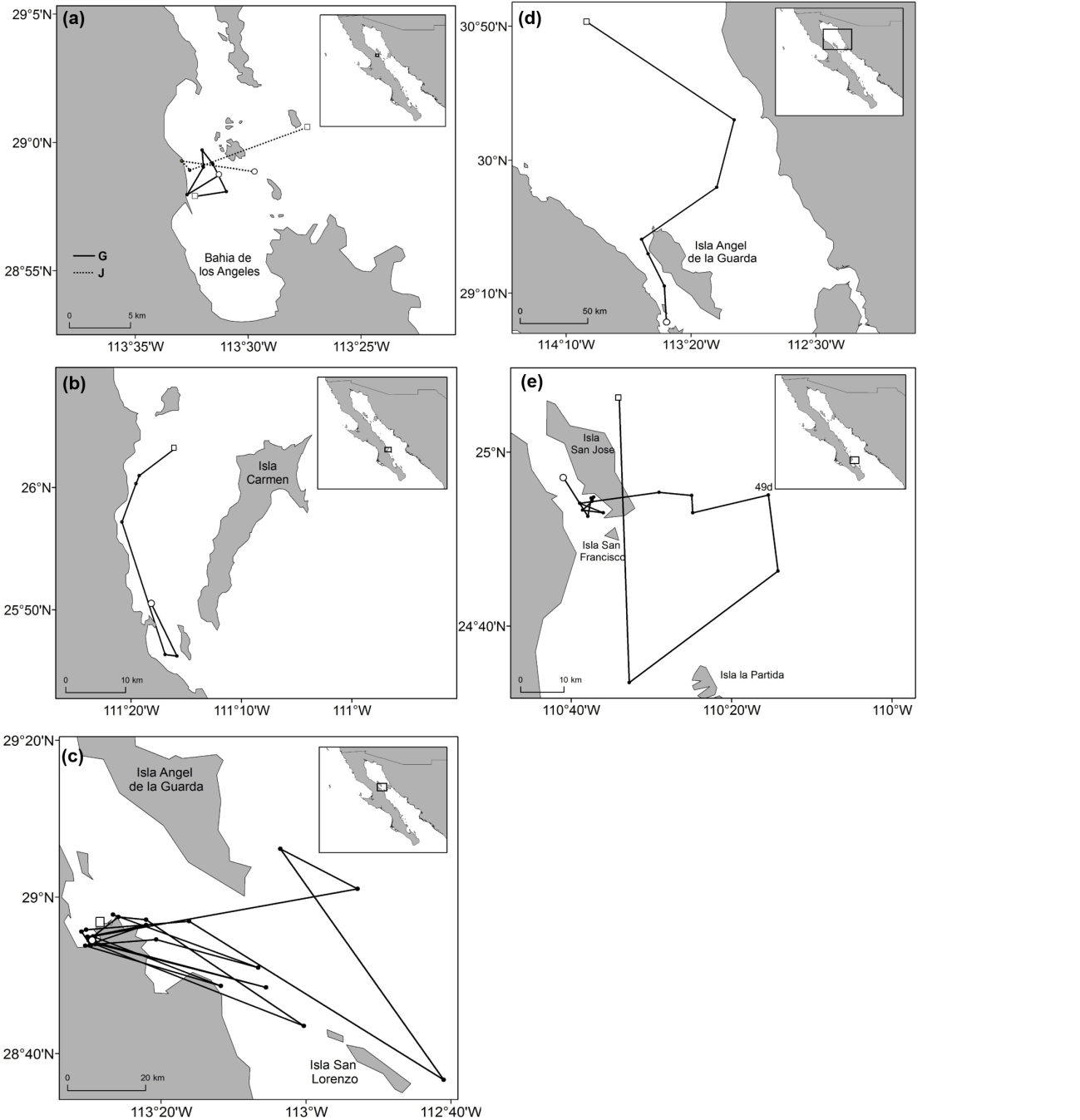

Supplement: S1 Fig — (a) movements of adult male Turtle G (Solid line) and an adult female (Dashed line) Turtle J in Bahia de los Angeles, Baja California. (b) Adult female Turtle L moving within the Bahia de Loreto, Baja California Sur. (c) Movements of adult male Turtle H moving out of Bahia de los Angeles to the south before returning to the area of its release. (d) Adult female Turtle K moving out of Bahia de los Angeles into the north of the Gulf of California and (e) adult male Turtle I foraging in the area of Isla San Jose and Isla Partida, Baja California Sur. (TIF) [file pone.0116225.s001.tif]

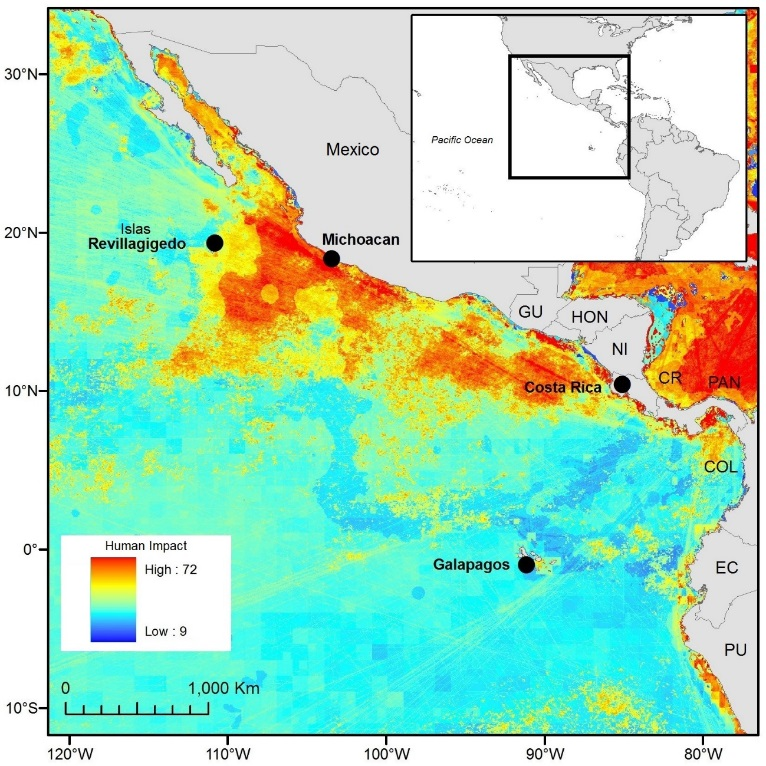

Supplement: S2 Fig — Black circles represent major green turtle rookeries in the East Pacific. Cumulative impacts: fisheries, pollution, invasive species, climate change, ocean acidification, nutrient input, human population pressure and commercial activities (shipping). (TIF) [file pone.0116225.s002.tif]
