# Supplementary material for: Multinational Tagging Efforts Illustrate Regional Scale of Distribution and Threats for East Pacific Green Turtles (Chelonia mydas agassizii)
Source: PLoS One. 2015 Feb 3;10(2):e0116225. doi: 10.1371/journal.pone.0116225 (PMC4315605; doi:10.1371/journal.pone.0116225)
Supplement: S1 Table — Flipper tag returns for turtles from the Michoacán, Alvarado & Figueroa [43]; Marquez & Carrasco [45] and Galapagos, Green [38] populations. (DOC) [file pone.0116225.s003.doc]

**Table S1.** Summary table of flipper tag returns by region for turtles from the Michoacán, Alvarado & Figueroa [1]; Marquez & Carrasco [2] and Galapagos, Green [3] populations.

|  |  | Michoacan | | Galapagos | |
| --- | --- | --- | --- | --- | --- |
| Country | State | Turtles recaptured (n) | Reference | Turtles recaptured (n) | Reference |
| Mexico | BC | 10 | Marquez & Carrasco [2] (n = 6)  Alvarado & Figueroa [1] (n=4) | - |  |
|  | BCS | 12 | Marquez & Carrasco [2] (n = 11)  Alvarado & Figueroa [1] (n=1) | - |  |
|  | Sonora | 12 | Marquez & Carrasco [2] (n = 11)  Seminoff et al. (2002) (n = 1) | - |  |
|  | Sinaloa | 11 | Alvarado & Figueroa [1] (n=3)  Marquez & Carrasco [2] (n = 7)  Zavala pers comm (n=1) | - |  |
|  | Nayarit | 9 | Marquez & Carrasco [2] (n = 9) | - |  |
|  | Islas Marias | 2 | Alvarado & Figueroa [1] (n=2) | - |  |
|  | Jalisco | 8 | Alvarado & Figueroa [1] (n=1)  Marquez & Carrasco [2] (n = 6)  Llamas pers comm (n = 1) | - |  |
|  | Colima | 3 | Alvarado & Figueroa [1] (n=3) | - |  |
|  | Guerrero | 16 | Alvarado & Figueroa [1] (n=1)  Marquez & Carrasco [2] (n = 15) | - |  |
|  | Oaxaca | 46 | Alvarado & Figueroa [1] (n=6)  Marquez & Carrasco [2] (n = 40) | - |  |
|  | Chiapas | 6 | Alvarado & Figueroa [1] (n=1)  Marquez & Carrasco [2] (n = 5) | - |  |
| Guatemala | - | 17 | Alvarado & Figueroa [1] (n=6)  Marquez & Carrasco [2] (n = 11) | - |  |
| El Salvador | - | 54 | Alvarado & Figueroa [1] (n=18)  Marquez & Carrasco [2] (n = 36) | - |  |
| Nicaragua | - | 3 | Alvarado & Figueroa [1] (n=2)  Marquez & Carrasco [2] (n = 1) | - |  |
| Costa Rica | - | 4 | Alvarado & Figueroa [1] (n=1)  Marquez & Carrasco [2] (n = 3) | 3 | Green et al. [3] |
| Panama | - | 2 | Marquez & Carrasco [2] (n = 2) | 4 | Green et al. [3] |
| Ecuador | - | - |  | 5 | Green et al. [3] |
| Peru | - | - |  | 10 | Green et al. [3] |
| Colombia | - | 3 | Marquez & Carrasco [2] (n = 2)  Alvarado & Figueroa [1] (n=1) | 1 | Green et al. [3] |

References

1. Alvarado J, Figueroa A (1992) Recapturas post-anidatorias de hembras de tortuga marina negra *Chelonia agassizii* marcadas en Michoacán, Mexico. Biotropica 24:560-566
2. Marquez & Carrasco (2002) The investigation and conservation of the black turtle in Mexico: the first years. In: Proceedings of the 22nd Annual Symposium on Sea Turtle Biology and Conservation, Miami FL 80-81
3. Green D (1984) Long-Distance Movements of Galapagos Green Turtles. J Herpetol 18: 2:121-130
